# Recent advances in hidradenitis suppurativa: Role of race, genetics, and immunology

**DOI:** 10.3389/fgene.2022.918858

**Published:** 2022-08-26

**Authors:** Gautham Vellaichamy, Anya T. Amin, Peter Dimitrion, Zaakir Hamzavi, Li Zhou, Indra Adrianto, Qing-Sheng Mi

**Affiliations:** ^1^ Center for Cutaneous Biology and Immunology Research, Department of Dermatology, Henry Ford Health, Detroit, MI, United States; ^2^ College of Medicine and Life Sciences, University of Toledo, Toledo, OH, United States; ^3^ Immunology Research Program, Henry Ford Cancer Institute, Henry Ford Health, Detroit, MI, United States; ^4^ Cancer Biology Graduate Program, School of Medicine, Wayne State University, Detroit, MI, United States; ^5^ Department of Internal Medicine, Henry Ford Health, Detroit, MI, United States; ^6^ Center for Bioinformatics, Department of Public Health Sciences, Henry Ford Health, Detroit, MI, United States

**Keywords:** hidradenitis suppurativa, genetics, immunology, ancestry, gamma-secretase complex, transcriptomics, proteomics, race

## Abstract

Hidradenitis suppurativa (HS) is a multifactorial chronic skin disease characterized by inflammation around the hair follicles commonly affecting intertriginous areas. The underlying pathogenesis of HS and its molecular mechanisms are largely understudied. Genetic studies in families have identified variants within the γ-secretase complex associated with HS; however, no definitive genotype-phenotype correlations have been made. The lack of knowledge regarding the intersection of genetics, immunology and environmental risk factors is a major obstacle to improving treatment for patients with HS. This article provides an overview of the role of race, genetics, and immunology in HS to provide insight into the multiple factors influencing the pathophysiology of HS.

## Introduction

Hidradenitis Suppurativa (HS) is a chronic, debilitating, inflammatory disease of the pilosebaceous unit characterized by painful nodules, abscesses, sinus tracts, and scar formation. HS is clinically under-recognized and mechanistically nebulous, leading to an average time to diagnosis of 7 years and has among the lowest patient-reported quality of life compared to other dermatologic conditions (Naik and Lowes, 2019; Hafner et al., 2021). HS has an extremely high comorbidity burden, rate of hospitalization, and recalcitrance to treatment, as well as disproportionately affects African Americans in both prevalence and severity (Reddy et al., 2019; Edigin et al., 2021; Sachdeva et al., 2021). Such observations, combined with the identification of monogenic variants of HS affecting racial minority groups, have rightfully led to studies addressing racial gaps in all aspects of HS research (Adelekun et al., 2020; Price et al., 2021). Recent immunological studies have uncovered new features about HS immunopathogenesis but have failed to account for the ethnic distribution and genetics of patients with HS (Navrazhina et al., 2021a; Navrazhina et al., 2021c). In this article, we provide an overview of the role of race, genetics, and immunology in HS based on recently published works in these fields. Here we utilize the definitions of race and ethnicity as put forth by the National Institutes of Health (NIH) guideline (NOT-OD-01-053) where both are considered as social and cultural characteristics as well as ancestry.

### Literature search methods

A comprehensive literature search was conducted in PubMed/Medline, Embase, and Google Scholar databases to identify publications on HS that 1) highlight recent (between 1/2019 and 5/2022) advances in treatments, genetic data, immunologic factors, or diagnostics and/or 2) report or discuss data on race of HS patients or study participants within the context of those same four categories. Database searches included the keywords “hidradenitis”, “suppurativa”, or “acne inversa”, in combination with one of the following: “race”, “ethnicity”, “genetics”, “immunologic”, “biologic”, “advances”, “systematic”, “meta-analysis”, “black/african american”, “caucasian/white/western”, “asian/eastern”, “pacific islander”, “hispanic/latino”, “south asian”, “middle eastern”, “european”. Boolean operators used included “AND” and “OR”. Citation review and handsearching of search results were then extensively performed to identify and stratify publications most relevant to the below subtopics. Reviews (including systematic reviews), meta-analyses, assessments of genotype-phenotype correlation, and studies that provided quantitative race data in regard to HS or HS research were prioritized for inclusion. Publications with minimal to no information on race within HS were excluded. In addition, studies that we felt were relevant to the mentioned topics but provided relatively weaker overall data were excluded given word count constraints. We then evaluated these studies within the context of recent advances in HS research and report our conclusions.

## Results

### Race representation in hidradenitis suppurativa studies is critical to unraveling its heterogeneous nature

Major efforts have been directed at spawning large-scale, racially-inclusive studies to more effectively probe the polygenic, multifactorial basis that is thought to underlie phenotypic heterogeneity and treatment failure in HS (Adelekun et al., 2020; Vellaichamy et al., 2021). At the clinical level, significant progress has been made to more accurately classify such disease heterogeneity (Frew et al., 2021b; Kirby, 2021), and outcome measures focused on patient-centered factors and clinically objective parameters have continued to be refined (Lyons et al., 2021). Notable efforts have been made in building national registries for patient data (Naik and Lowes, 2019; Adelekun et al., 2020) and clear protocols have actively been developed to use these data in conjunction with surgically obtained tissue specimens (“biobanking”) for molecular studies (Byrd et al., 2019).

While the presence of numerous confounders and inherent differences in sampling have made true prevalence estimates difficult, systematic reviews reveal an average prevalence of HS of 1.3% in African Americans, 0.75% in Caucasians, 0.07% in Hispanics, 0.17% in other races (Sachdeva et al., 2021). Targeted prevalence estimates in Taiwan and Korea demonstrate disease burdens of 0.2% and 0.1%, respectively (Van Straalen et al., 2022). Among Phase II/III clinical trials on HS between 2000 and 2019 only 60% of studies published demographic data, and the majority of trials occurred in the United States and Europe (68% Caucasian, 14% African, 2.9% Asian, and 0.4% American Indian) (Price et al., 2021). Among genetic studies, African Americans are severely under-represented despite reporting equally high rates of family history (Vellaichamy et al., 2021). In addition, a recent meta-analysis focusing on East and Southeast Asian populations notably lacked randomized-controlled trials but did include several cross-sectional cohorts and case-based studies. In contrast to HS in the Western regions, in these populations, HS tended to preferentially affect males, the axillary and gluteal regions, and report a lower rate of positive family history (5 versus 30%) (Gotesman et al., 2021). While environmental variables including smoking and obesity are proposed explanations for these differences, underrepresentation of these populations in genetic studies is likely to be a significant contributor, given that a large proportion of available targeted genetic studies on familial HS are described in Asian patients (particularly γ-secretase mutations) (Van Straalen et al., 2022). Notably, minimal studies have demonstrated the presence of such mutations in Western populations, which is likely due to both underrepresentation of Western familial cohorts as well as yet to be determined hereditary differences among ethnic groups (Vellaichamy et al., 2021; Van Straalen et al., 2022). Furthermore, a study in Japanese HS patients demonstrated similar male predominance, a lower rate of family history (4%) and a higher relative incidence of Hurley Stage II/III disease (Hayama et al., 2020). Phenotypic differences among Western patients in comparison with Asian patients also exist. The groin, followed by axillae, perianal and perineal regions were the most frequently affected areas in Europe, whereas the buttocks were the most prevalent areas in Korean and Japanese patients (Chandran et al., 2021; Van Straalen et al., 2022). Such studies highlight that complex genetic architecture may be contributing to phenotypic differences in ethnically diverse groups.

### Genetics remains an area of need in the study of hidradenitis suppurativa

The genetic contributions in HS, driven by heterozygous, nonsynonymous mutations in the γ-secretase complex, have been identified in several studies as a monogenic variant that co-segregates with disease phenotype and with high penetrance (Wang et al., 2010). Such variants were found in nearly every race, with most in minority populations (including Asian and Middle-Eastern, among others) exhibiting atypical, severe, or syndromic disease with significant heritability across generations (Lowe et al., 2020; Vellaichamy et al., 2021). A large-scale twin study in a Danish population demonstrated heritability estimates of up to 80% (Kjaersgaard Andersen et al., 2021). More recent work has pushed genetic studies to include larger sample sizes and explore multiple-gene-related mechanisms with race as a key variable (Kjaersgaard Andersen et al., 2021; Theut Riis et al., 2021; Vural et al., 2021); however, no GWAS studies identifying disease-associated single nucleotide polymorphisms have been done, limiting the extrapolation of this data. Such studies are paramount, as polygenic, gene-gene interaction-based mechanisms likely outweigh monogenic contributors in the overall burden of HS (Kjaersgaard Andersen et al., 2021).

### Biologics are promising therapeutic options for hidradenitis suppurativa patients

Biologic medications have been at the forefront of clinical investigations given their demonstrated promise in recent years in tempering and controlling recalcitrant HS. Adalimumab (Humira), the only FDA-approved biologic for HS, was shown to be effective for approximately 50% of patients in two watershed phase III trials (Kimball et al., 2016) and this improvement was sustained through 168 weeks (Zouboulis et al., 2019). Real-world efficacy of adalimumab, infliximab, and infliximab-abda (Intravenous route) have been recently assessed in 1-year studies (Gulliver et al., 2021; Hafner et al., 2021; Marzano et al., 2021; Westerkam et al., 2021). The variable response to biologics has been an intense area of focus within pharmacogenomics, particularly via immunogenetics studies seeking to identify predictive biomarkers of treatment response and disease progression as well as other pharmacologic targets (Frew et al., 2021c). Examples include a genome-wide association study of Pioneer I/II trials showing a BCL2 variant associated with response to adalimumab in patients with HS in a TNF-dependent matter localized to the follicular unit (Liu et al., 2020). Polymorphisms in the TNF-gene were also found to be significantly associated with disease susceptibility and disease course of HS (Savva et al., 2013). Other biomarkers may include HCC-4, calprotectin, and fractalkine (Cao et al., 2021). Race-stratified data, however, is sparse within these studies, and given the majority of trials taking place in the United States and Europe, non-Caucasian groups are notably underrepresented (Price et al., 2021).

### Transcriptomic and proteomic studies have further characterized the distinct, inflammatory nature of hidradenitis suppurativa

Modern-day efforts in HS research are actively moving in the right direction, and at a faster rate than ever before. Multi-institutional efforts to develop long-term, large-scale data repositories in conjunction with specimen analysis are key steps to unraveling genotype-phenotype correlation in HS (Byrd et al., 2019; Frew et al., 2019). Immune dysregulation lies at the heart of HS pathogenesis and recent studies into the transcriptome and proteome have pointed to local and systemic inflammation as features of HS, which might explain specific disease features (Navrazhina et al., 2021a; Navrazhina et al., 2021c). These studies highlight the importance of integrative approaches to study HS, wherein peripheral and cutaneous immune dysregulation are resolved jointly. The increasing number of different immunological pathways posited to play a role in HS inflammation suggests that multiple immunophenotypes of HS exist (Gudjonsson et al., 2020; Lowe et al., 2020; Brandao et al., 2022). Advanced techniques including single-cell RNA-sequencing and Cytometry by Time-of-Flight (CyTOF) enable us to unravel complex immunological interactions and identify discrete disease subtypes and novel pathways propagating disease. A better understanding of the relationship between immunopathogenesis and clinical outcome will support efforts to develop precision medicine-based treatment and improve prognostication and clinical management for HS.

Recent immunological studies have focused on transcriptomic and proteomic analysis of biospecimens (i.e, serum, skin biopsies) to characterize the inflammation in HS, which is known to extend beyond lesional to perilesional and non-lesional skin, spurring refinement of anatomic definitions in clinical research. RNA sequencing of site-matched untreated HS versus control demonstrated that high and low inflammatory sub-phenotypes of HS could be distinguished by levels of lipocalin-2, a neutrophil- and IL-17- associated marker, (Navrazhina et al., 2021c). In addition, a large-scale proteomic analysis of HS patient serum showed that high lipocalin-2 levels were associated with the presence of dermal tunnels as well as clinical severity in patients (Navrazhina et al., 2021b). Dermal tunnels and deep dermal inflammation are key histopathologic features distinguishing HS from other systemic inflammatory dermatoses, and thus these key studies shed insight into yet to be understood mechanisms. Two proteomic studies comparing HS with psoriasis found a greater serum inflammatory burden, a more TH_1_/TH_17_-skewed cytokine profile, and a high TNF-α signature in comparison with psoriasis (Navrazhina et al., 2021d). IFN-γ, IL-36, and TNF-α, as well as B-cells and plasma cells were also shown to be players in HS-related inflammation (Gudjonsson et al., 2020; Lowe et al., 2020). The serum-skin correlation was also investigated; CSF3 mRNA levels in lesional skin significantly correlated with neutrophil-related proteins in the serum, implicating CSF3 as a possible driver of neutrophilic inflammation in the skin. While efforts continue, relatively small sample sizes linger as an issue in HS research. The identification of a broad immune dysregulation implies that specific endotypes of HS exist, which rely on discrete inflammatory mechanisms. Dissecting this heterogeneity could prove useful in predicting better response to the growing list of biologic therapeutics employed to treat patients with HS.

Investigating the causes of racial disparities in the prevalence and disease severity of HS is critical to helping researchers develop new treatments and prevention strategies for patients with HS that have the worst outcomes. A recent study comparing the transcriptomes of skin biopsies from healthy African Americans and Whites revealed the presence of proinflammatory genes such as TNFα, IL-32 in African American skin, and keratinocytes that may contribute to the increased sensitivity or risk of African Americans to the development of HS (Klopot et al., 2021). This study also found that differentially expressed genes in African American skin and keratinocytes overlapped with atopic dermatitis and psoriasis gene signatures. These differentially expressed genes may also be enriched in African American HS patients, but studies comparing the transcriptomes of HS patients from different ethnic backgrounds has not been done. While the underlying mechanisms of how these genes increase the risk for specific skin diseases remain unclear, identifying differences in the initiating factors that are responsible for the development of HS in patients with distinct genetic backgrounds is needed for the development of novel targeted therapeutics.

Adjunct diagnostic modalities should continue to be studied, such as ultrasound which can utilize parameters like epidermal thickness and doppler power intensity that are shown to correlate with the quality of life and pain scores, respectively (Frew et al., 2021a). All studies, and in particular clinical trials, should continue to make ample effort to include proportionally representative populations of racial sub-groups.

## Conclusions and perspectives

Despite advances in HS research, specific genotypic and epidemiologic factors that contribute to HS risk are poorly understood and the lack of mechanistic understanding is a principal barrier to improving treatments for HS. While identified monogenic, hereditary forms of the disease (particularly in patients with γ-secretase mutations) have driven some understanding into this process, the majority of disease burden is indeed sporadic with the majority of patients reporting negative family histories. Thus, the lack of genome-wide and other large scale studies investigating gene-gene interactions is the prime barrier to the next level of understanding the genetic basis of HS.

The difference in the prevalence of HS by race is striking. While the majority of studies occur in Caucasian populations, African Americans have established prevalence and incidence rates that are approximately 3-fold higher than European Americans (Garg et al., 2017a; Garg et al., 2017b). Further, African American patients exhibit higher disease severity and the greatest number of comorbid conditions (Lee et al., 2017). Genetic studies accounting for admixing of different ethnic populations will be necessary to understand the genetic architectures of HS patients from different ancestries. Understanding the nuances in the genetic landscape of HS will reshape our understanding of HS pathophysiology. It is plausible that differences in African ancestry among African Americans may underlie their increased HS risk.

**FIGURE 1:**
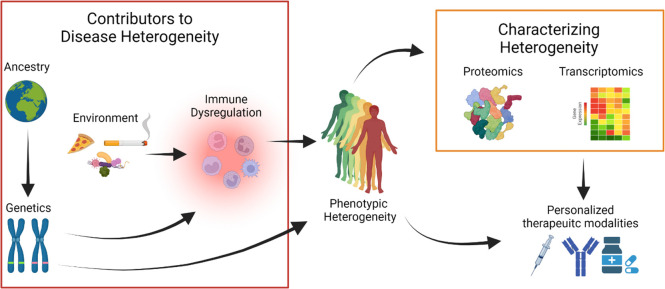
Hidradenitis Suppurativa (HS) is a phenotypically heterogeneous disease. Clinical studies of patients from different ancestries define discrete disease manifestations pointing at a complex genetic architecture. Genetic perturbations may influence immune responses or have other mechanisms that directly explain disease heterogeneity. Environmental exposures may also contribute to disease heterogeneity, mediated by perturbations in the immune system. Proteomic and transcriptomic technologies can reveal disease heterogeneity by defining specific protein and gene signatures that are associated with distinct HS phenotypes. In combination with clinical data, multi-omics data can help define a framework to use personalized therapeutic modalities from the growing arsenal of HS treatments.

The multi-factorial etiology of HS makes dissection of causative features of disease challenging. Both intrinsic and extrinsic factors likely contribute to phenotypic heterogeneity. Discussed above, patients with HS from different ancestries have different clinical presentations. While the role of extrinsic factors (i.e., smoking, diet, microbes) have been strongly linked to immune dysregulation, the dearth of genetic information leaves a huge gap in determining how these intrinsic and extrinsic factors jointly contribute to dysregulation of the immune system. In addition, future studies utilizing proteomics and transcriptomics to characterize the molecular basis of this clinical heterogeneity could develop new models of treatment to better use a growing arsenal of biologics and other therapeutic modalities (Figure 1).
